# Development and validation of a non-invasive method for quantifying amino acids in human saliva†

**DOI:** 10.1039/d4ra01130a

**Published:** 2024-07-15

**Authors:** Md. Mehedi Hasan, Mamudul Hasan Razu, Sonia Akter, Salma Akter Mou, Minhazul Islam, Mala Khan

**Affiliations:** a Bangladesh Reference Institute for Chemical Measurements Dhaka Bangladesh dg.mic@bricm.gov.bd mhrazu@bricm.gov.bd

## Abstract

As an analytical matrix, saliva has superior characteristics than blood and urine. Saliva collection is, first and foremost, non-invasive, making it convenient, painless, and secure for more susceptible people. Second, it does not need professional training for medical personnel, resulting in cost-effectiveness and suitability for extensive collection in support of research. In this study, we developed a method and used it to quantify 13 salivary-free amino acid (SFAA) profiles to support the early clinical diagnosis of diseases using LC-MS/MS. Using an Intrada Amino Acid column (100 × 3 mm, 3 μm), chromatographic separation was accomplished with a binary gradient elution, and an electrospray ionisation source running in the positive ionisation mode was chosen for data collection using the multiple reaction monitoring (MRM) modes. Amino acids were extracted from saliva using acetonitrile. In the MRM mode, LODs and LOQs for ten amino acids were in the range of 0.06–2.50 μM and 0.19–7.58 μM, respectively, and those values were in the range of 1.00–3.00 μM and 3.00–8.50 μM, respectively, for three amino acids. Matrix-matched six-point calibration curves showed a linear correlation coefficient (*r*^2^) of ≥0.998. Recovery experiments validated the method by spiking the control sample at three different concentration levels (5, 50 and 100 μM), and the accuracy level was 85–110%. Except for Thr and Ser, intra- (*n* = 3) and inter-day (*n* = 3) precision fell between 0.02 and 7.28. Salivary amino acids can serve as possible biomarkers for various malignancies, with fluctuations in body fluids being crucial for cancer diagnosis; therefore, examining amino acid patterns in saliva can assist in early cancer detection. LC-MS offers improved selectivity and sensitivity for non-derivatised amino acid analysis, surpassing conventional methods and offering proactive quality assurance, making it suitable for complicated sample matrices. These discoveries could be significant in investigating new pathways and cancer treatments and looking for possible AA biomarkers for other malignancies and diseases.

## Abstract 2

As an analytical matrix, saliva has superior characteristics than blood and urine.

## Graphical abstract

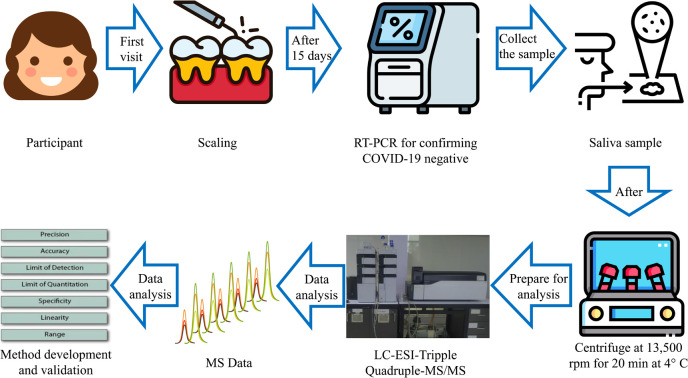

## 1. Introduction

Many studies have demonstrated that different types of cancer may have distinct amino acid biomarkers and that differences in the amount of amino acids in body fluids and tissues are essential for both cancer diagnosis and treatment plan selection.^1–3^ Saliva is one of the most suitable bodily fluids for amino acid analysis.^4–12^ Salivary amino acid profiling has immense potential as a non-invasive diagnostic technique in cancer research.^13^ Research has demonstrated that salivary amino acids can potentially serve as biomarkers for different cancers, and variations in amino acid concentrations in body fluids play a crucial role in cancer diagnosis.^14^ Analyzing amino acid patterns in saliva can help doctors enhance early diagnosis, prognosis, and patient outcomes, as well as contribute to the development of customised medicines.^15,16^ Sugimoto *et al.* used CE-TOF-MS to analyse saliva samples collected from breast cancer patients, and they identified 28 salivary metabolites for breast cancer, of which 14 AAs had significant values.^17^

Amino acids, fundamental components of all biological activities, function as sensors in signaling networks. Amino acids supply protein substrates, help in nucleic acid production, and engage in carbohydrate and lipid metabolism. The majority of amino acids serve as the building blocks of proteins and are crucial for preserving the equilibrium of several fundamental processes such as hormone secretion,^18^ regulation of metabolism,^19^ immune response,^20^ and expression of genes.^21^ Additionally, they participate in epigenetic changes (mainly through *S*-adenosylmethionine's involvement as a donor of methyl groups) and antioxidant mechanisms that are not enzymatic (by glutathione synthesis).^22–24^ Early diagnosis leads to a higher survival percentage with potential treatments in most cases of different diseases. For the last ten years, saliva has been clinically used as a diagnostic tool, drawing much attention and establishing itself as a reliable technique.^14^ Therefore, amino acid profiling is crucial for examining metabolic dysregulation and regulation.^25^

The diagnosis and treatment of a wide range of metabolic disorders, such as phenylketonuria, maple syrup syndrome, and cystinuria, as well as assessments of tissue damage, renal function, dietary compliance, and nutritional status, all depend heavily on the quantitative evaluation of free amino acids in biological fluids such as saliva and plasma.^26^

A growing number of clinical areas are using salivary biomarkers to monitor and identify diseases, including Sjögren's syndrome, colorectal cancer, pancreatic cancer, thyroid cancer, liver cancer, gastric cancer, lung cancer, glioblastoma, periodontal disease, breast cancer, and oral squamous cell carcinoma. In recent years, the application of biomarkers to early clinical disease prediction has also improved the assessment of potential health risks.^13,27–31^ Researchers have demonstrated that cancer cells have a markedly different metabolism of AAs,^32^ and there is a significant difference between cancer patients and people with no cancer in terms of plasma-free amino acids (PFAA).^33–35^

As documented, insulin resistance can cause significant disruptions to amino acid patterns.^36^ Besides posing a growing hazard to public health, metabolic profiles for diabetes, metabolic syndrome, and obesity aim to advance our understanding of these conditions' etiology and treatment approaches. Additionally, amino acid analysis is a crucial analytical tool for many medicinal, pharmacological, and agricultural applications and metabolic and metabolomic research studies.^37,38^ Various techniques have been proposed for analysing amino acids since it is still challenging to quantify them from multiple complex biological matrices efficiently and thoroughly.^25^

While the amino acid analyser is currently considered a standard technology for diagnostic purposes, its lengthy 120 minute run time and consequent high overall costs per sample are significant drawbacks.^39^ This new method offers comprehensive salivary-free amino acid profiling within a short analysis time while using non-derivatised amino acids.^40^ Many laboratories use derivatisation (*e.g.*, aTRAQ, AQC, bromobutane) to aid retention and separation.^41^ They are similar to other derivation methods but increase possible errors, imprecision, sample complexity, and sample preparation time (10–60 min). A non-derivatised amino acid analysis approach would be preferable for many labs looking for simplicity and cost savings.^42^ Analytical challenges persist in inefficiently and comprehensively assessing amino acids from complex biological matrices. Researchers have developed numerous semi-automated techniques for analysing amino acids in industrial and clinical settings. Recently, publications have detailed methods for utilising liquid chromatography-tandem mass spectrometry (LC-MS/MS) to analyse amino acids in physiological samples.^43^ LC-MS provides improved selectivity and sensitivity for non-invasive salivary amino acid analysis, exceeding current methods.^44^ It offers proactive quality assurance (QA) beyond physical or instrument tests and is ideal for complex sample matrices.^45^

This study aimed to develop a rapid, precise, and accurate LC-MS/MS method for real-time, intended analyses of underivatised amino acids in saliva samples. Hydrophilic interaction chromatography was used for separating the amino acids for all analytes, improving retention and peak symmetry. This approach successfully validated the simultaneous evaluation of AAs in a 400 L human saliva sample. In Bangladesh, for the first time, researchers of BRiCM have made an unprecedented find by studying the amino acid profile of human saliva. This pioneering finding sheds light on a non-invasive way of disease detection. These findings could help investigate novel pathways and cancer treatments and search for potential AA biomarkers for other forms of carcinoma and diseases.^46^

## 2. Method and materials

### 2.1 Chemicals and reagents

Following reagents with corresponding suppliers were used in the experiments: standard amino acid solution (Analytical, Sigma Aldrich, Germany), acetonitrile (MS grade Honeywell, Germany), ammonium formate (98%, Merck, Germany), tetrahydrofuran (≥99%, Honeywell, Germany), formic acid (≥98%, Merck, Germany), and methanol (MS grade Honeywell, Germany).

### 2.2 Stock solution and intermediate stock solution preparation

2500 μM of the amino acid standard solution was prepared in methanol and water (50 : 50). After sonicating for a minute, the solution was kept cold (4 °C) to facilitate the subsequent production of calibration solutions. Methanol and water (50 : 50) were used to dilute stock solutions appropriately to prepare the intermediate stock solutions of 2.0 μM, 5.0 μM, 10 μM, 20 μM, and 50 μM of amino acids.

### 2.3 Saliva sample preparation

Healthy volunteers provided human saliva during the first visit, and dental scaling was performed. We followed up with each participant over the phone after 15 days, at which point their periodontal health had improved, and we ran another RT-PCR test. After establishing that COVID-19 was negative, we collected the unstimulated saliva between 9:00 and 11:00 a.m., the next day. Before collecting their saliva, the subjects were told not to eat for two hours. They received instructions to rinse their mouths with water for ten minutes, sit straight, lean their heads slightly forward, and spit at least 5 ml of saliva into a calibrated test tube.

After being collected, the samples were centrifuged (Korea) for 10 minutes at 4 °C at 10 000 rpm to eliminate insoluble materials, food residue, and cell debris. The resultant supernatants were frozen at −80 °C until the lab analysis. Then, in a 2.0 ml Eppendorf tube, 400 μL of the thawed saliva aliquots were mixed with 800 μL of acetonitrile, and the mixture was vigorously shaken for one minute to precipitate the proteins. After standing for 15 minutes, the mixture was centrifuged for 20 minutes at 4 °C at 10 000 rpm. Following filtration through 0.22 μm syringe filters, the supernatant was ready for LC-MS/MS analysis.

### 2.4 Analytical conditions

LC-MS/MS analysis utilised a Shimadzu Ultra-Fast Liquid Chromatography System (8050, Shimadzu Corporation, Kyoto, Japan) equipped with an electrospray ionisation (ESI) source, an autosampler, binary pumps, and a column oven. This system was coupled to a triple-quadrupole mass spectrometer. We applied an optimised gradient elution program using a unique combination mode for thirteen genetically encoded amino acids. The following explains the specifics of the LC and MS conditions. A 100 × 3 mm, 3 μm column kept at 35 °C for amino acid analysis was used. The mobile phase consisted of a mixture of two solutions (A: ACN/THF/25 mM ammonium formate/formic acid: 9/75/16/0.3 and B: ACN/100 mM ammonium formate: 20/80) with a gradient elution program that included the following steps: 0% B (0–3.0 min), 0–17% B (3.0–9.0 min), 17–100% B (9.0–16.0 min), 100% B (16.0–22.0 min), and 0% B (22.0 min). The flow rate was 0.6 mL min^−1^. The chromatographic injection volume was 10 μL, and the retention time of amino acids was approximately 22 minutes. The following were the MS acquisition parameters: run time: 22 minutes; ion source: atmospheric pressure electrospray ionisation; ion polarity: positive ion mode; block temperature: 400 °C; desolvation line temperature: 300 °C; capillary voltage (kV): 4.0; argon (270 kPa) is the CID gas; 1.5 L min^−1^ is the nebulising gas; 15.0 L min^−1^ is the drying gas; 10 L min^−1^ is the heating gas; and 300 °C is the interface temperature.

### 2.5 Method validation

According to the International Conference on Harmonisation's recommendations,^28^ the LC-MS/MS method was validated using several factors, including sensitivity, accuracy, recovery, precision, linearity, specificity, and selectivity.

#### 2.5.1 Sensitivity

Expressing the lowest non-zero value of a particular analyte as LOD and LOQ, we identify LOD as the lowest detectable concentration of the target analyte. At the same time, LOQ signifies the lowest quantifiable concentration with satisfactory precision. Determining these values involves assessing the signal-to-noise ratio (S/N) from the relevant blank. Computation of the LOD and LOQ for amino acids relies on this ratio using % RSD. Establishing the limits of quantification (LOQ) entails considering the lowest calibration level for each amino acid capable of producing a signal of the qualifier ion in the correction ratio. Based on these parameters, the peak intensities had to have S/N > 3.

#### 2.5.2 Linearity

Linearity testing occurred at five concentration levels: 2.0 μM, 5.0 μM, 10 μM, 20 μM, and 50 μM. Plotting the peak area *vs.* concentration (μM) led to a calibration curve. For every amino acid examined, Table 2 illustrates that the *R*^2^ and regression coefficient values obtained from the calibration curves were higher than 0.999, suggesting good linearity for the approach for each of the thirteen analytes under investigation.

#### 2.5.3 Accuracy

To calculate the accuracy, the standard proportion of the measured concentration to the actual at three different levels, STD-L (5.0 μM), STD-M (20.0 μM), and STD-H (50.0 μM), was applied for six replicates in each level.

#### 2.5.4 Recovery

Analytical method recovery is the percentage of the calculated value found by the method closest to the analyte's nominal concentration. Samples spiked using established concentrations of the standard and control solutions were used to evaluate method recovery. Recovery, indicating the percentage of the analyte remaining after adding a known amount to a sample, was assessed using three concentration levels. These levels corresponded to 20%, 50%, and 80% of the total working range. To determine the average recoveries, we applied the formula: recovery (%) = [(amount found-original amount)/amount added] × 100. Recovery experiments were carried out at four concentrations in order levels, with three replicates each on two days.

#### 2.5.5 Precision

Six replicates of the sample with spiked low, medium, and high concentration levels of the targeted analytes throughout the working range and guidelines were analysed to determine the method's precision.^47,48^ The precision was evaluated under reproducible conditions by multiple analysts on separate days (known as inter-day precision) and under repeatable conditions by the same analyst on the same day (known as intra-day precision). The same analyst evaluated the method's intermediate precision on the same day (intra-day precision) under repeatable circumstances, and other analysts reviewed it in random order on separate days (inter-day precision).

Three different concentration levels Spike-L (5.0 μM), Spike-M (20.0 μM), and Spike-H (50.0 μM) of mixed standards were employed to evaluate the procedure's repeatability. The repeatability was measured using the sample's relative standard deviation (RSD%) and mixed standard peak regions for six replicates at three different concentrations, respectively.

#### 2.5.6 Specificity and selectivity

A method achieves selectivity when its response is distinct from all other responses. That means the analytical procedure should distinguish the endogenous components of the matrix containing the analytes of interest and other elements in the sample.

### 2.6 Statistical analysis

A two-way ANOVA with Bonferroni post-tests was employed to examine differences in saliva amino acid levels (Prism, GraphPad Software Inc., USA, and R). A value of *P* < 0.05 was deemed statistically significant.

## 3. Result and discussion

The components of saliva and blood are relatively similar. Even though the salivary glands can only produce a certain amount of molecules, the molecules left in saliva must have particular characteristics for the blood to transfer into saliva. Among the different types of transportation are extracellular and intercellular routes, ultrafiltration, passive diffusion,^49^ and active transport.^50^ Saliva is a biological matrix with potential applications for low-cost, non-invasive analysis; it is especially well-suited for gathering vast amounts of data for investigations and clinical trials. Cancer cells consume higher amounts of amino acids and glucose than their benign counterparts. As peripheral proteins break down, amino acids are released and transported to the visceral organs and tumors. These amino acids may be useful in the development of tumours, the gluconeogenic pathway, and cell division.^51^ Studies have shown that amino acids and their derivatives are useful markers of the metabolism of proteins. Increased quantities of amino acids in saliva may serve as a signal for a particular disease.^52^

Numerous researchers have reported that AA metabolism is markedly altered in cancer cells^32^ and that there is a considerable difference in plasma-free amino acids (PFAA) between cancer patients and healthy controls.^33–35,53^ These findings align with earlier research on the profile of plasma-free amino acids. Cascino *et al.* demonstrated a significant increase in plasma levels of free tryptophan, glutamic acid, and ornithine.^54^ Significantly higher amounts of Thr, Pro, Gly, and Ala (*P* < 0.001); Ser, Orn (*P* < 0.01), and Lys (*P* < 0.05) were found in breast cancer patients, according to Miyagi *et al.*^35^ Patients with breast cancer had noticeably higher amounts of Thr, Ser, Glu, and Orn, according to Naoyuki *et al.*^53^ Research has demonstrated notable alterations in the AA profiles of cancer patients' bodily fluids, indicating that variations in free AAs stem from cancer-specific modifications in AA metabolism. Early studies hypothesised that starvation was the reason behind the alteration of AA profiles in cancer patients. Anorexia may cause cancer patients to lose weight by reducing their food intake.^55^ Cancer patients who are malnourished have elevated protein synthesis and metabolism, putting them in a hypermetabolic condition.

Nonetheless, there is ongoing debate and ambiguity over the mechanism underlying the aberrant AA profiles in cancer patients. According to early studies,^56^ AA profiles were exclusively aberrant in cancer patients who had lost weight. However, subsequent studies^33,35,53^ have shown that cancer patients who did not lose weight still had aberrant AA profiles. According to earlier studies,^34,35,53^ plasma-free AA profiles often rise in breast cancer but sharply decline in digestive organ malignancies; this may be because breast cancer typically does not grow as quickly or aggressively as other metabolically active malignancies.^57^ The study's findings also demonstrated a relationship between the kind of tumor and aberrant AA profiles, indicating that tumors originating from various organs had distinct AA profiles. Malnutrition may affect the aberrant AA profiles observed in cancer patients. Furthermore, the kind and stage of the cancer can be far more significant variables.

### 3.1 Method development and optimisation

Due to the variety of their hydrophilic qualities and zwitter ionic characteristics, simultaneously determining all 20 endogenous AAs chosen for this investigation is highly challenging. That is why real-time analysis of AAs is challenging because of limited retention on a reverse phase column and the isomeric characteristics of AA, which is another important obstacle for MS/MS analysis. In this work, we used various methods to overcome the difficulties and improve the mass spectrometric and chromatographic settings. ESI was employed to ionise all analytes, and each AA analyte's precursor and product ions were identified through stepwise collision energy in the MRM mode and full scan MS analysis. For each MRM transition, the collision energy (CE) and clustering potential (DP) were manually adjusted by injecting the reference compounds using an autosampler. Utilising the extracted saliva sample and the standard AAs mixed solution, various mass spectrometric parameters were adjusted individually, such as ionisation voltage, interface temperature, nebuliser gas flow, and drying gas flow. Using an Intrada Amino Acid column to optimise LC conditions and various stationary phases revealed a satisfactory retention time of certain highly polar compounds over a range of conditions.

**Fig. 1:**
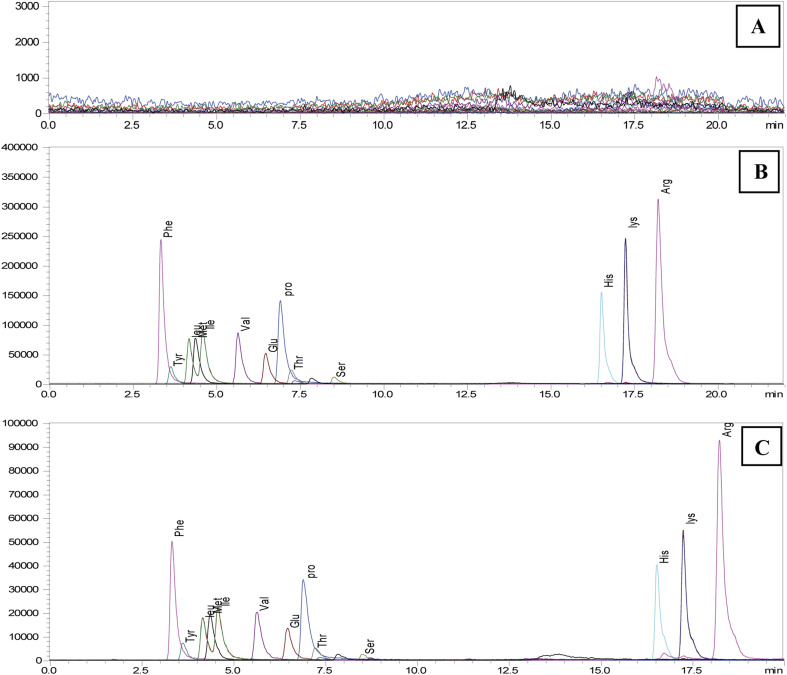
Simple mass chromatogram of 13 amino acids of (A) blank, (B) spike-100 μM concentration and (C) STD-100 μM concentration using LCMS/MS system.

Additional optimisation was performed to enhance sensitivity and chromatographic performances of various AAs by altering the types of mobile phases and the amounts of organic solvents and additives. Adding ammonium formate to enhance elution capacity and incorporating formic acid resulted in the production of more signals. Fig. 1 displays a simple mass chromatogram featuring 13 amino acids. Solution A comprised acetonitrile, tetrahydrofuran, 25 mM ammonium formate, and formic acid (9 : 75 : 16 : 0.3). Solution B contained 100 mM ammonium formate dissolved in water. The isomers Leu (3.26 min) and Ile (3.51 min) reached baseline separation. Additionally, a SIM mode could be used to analyse glycin. With a total run time of 22 minutes, 13 natural AAs with a retention time shift of less than 0.2 minutes and their related derivatives were satisfactorily retained and separated by the chromatographic performance.

### 3.2 Method validation

According to the International Conference on Harmonization's guidelines,^28^ the LC-MS/MS method is validated using several factors, including robustness, LOD, LOQ, specificity, linearity, precision, and accuracy.

#### 3.2.1 Sensitivity

The excellent sensitivity of the MS detector for the identification, which can detect trace quantities, was made possible by the computed LODs and LOQs for the amino acids, which were in the ranges of 0.06–2.50 μM and 0.19–7.58 μM, respectively. Whereas, Cheng *et al.* reported their LOD and LOQ in the ranges of 0.006–0.14 μM and 0.02–0.47 μM, respectively,^27^ and our data are closely related. Also, Qu *et al.* reported their LOD and LOQ in the ranges of 0.1–5 μM and 0.2–0.10 μM, respectively,^58^ where our LOD and LOQ were much lower. Except for Thr and Ser, this ratio allowed for determining the LOD and LOQ for amino acids with a % RSD of less than 5. Table 1 shows that Lys had the lowest LOD and LOQ values, whereas Ser had the highest LOQ values.

**Table 1:** Optimised detection limits and MS conditions for individual AA analyses on Shimadzu-8050

Name	Ret. time	Unit	*m*/*z*	S/N ratio	LOD	LOQ
Phe	3.354	μM	166.10 > 120.10	24.62	0.25	1.87
Tyr	3.657	μM	182.10 > 136.20	6.81	0.85	1.752
Leu	4.132	μM	132.10 > 86.30	7.98	0.66	1.602
Met	4.397	μM	150.10 > 56.10	39.66	0.13	1.619
Ile	4.542	μM	132.10 > 86.30	10.49	0.58	1.841
Val	5.628	μM	118.20 > 72.00	10.64	0.63	2.047
Glu	6.594	μM	148.10 > 84.10	11.44	0.5	1.746
Pro	6.944	μM	116.10 > 70.10	15.01	0.43	1.938
Thr	7.233	μM	120.10 > 74.00	4.63	1.43	1.999
Ser	8.445	μM	106.10 > 60.20	2.79	2.23	1.882
His	16.037	μM	156.10 > 110.10	19.61	0.33	1.986
Lys	16.67	μM	147.00 > 84.10	105.34	0.06	1.958
Arg	17.609	μM	175.10 > 70.10	65.31	0.11	2.208

**Table 2:** An overview of the linearity and calibration range of the combined MRM-SIM technique for amino acids on the Shimadzu-8050 instrument

Amino acid (100 μM)	Linearity (*R*^2^)	Calibration range (μM)
Phe	0.9999	2–50
Tyr	0.9993	2–50
Leu	0.9997	2–50
Met	0.9993	2–50
Ile	0.9993	2–50
Val	0.9988	2–50
Glu	0.9997	2–50
Pro	0.9982	2–50
Thr	0.9973	2–50
Ser	0.9983	2–50
His	0.9997	2–50
Lys	1.0000	2–50
Arg	0.9998	2–50

#### 3.2.2 Linearity

**Fig. 2:**
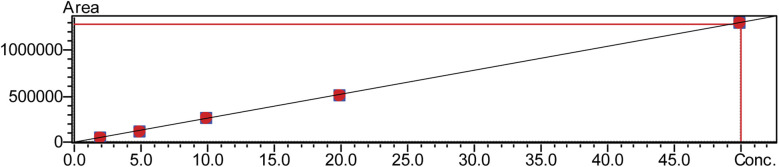
Calibration curve of phenylalanine of 5 different concentration points using the LCMS/MS system.

The following analysis was performed using advanced software (Lab Solutions Insight). The calibration range had five calibration levels, which were 2.0 μM, 5.0 μM, 10 μM, 20 μM, and 50 μM. We used line regression to create the calibration curves, matching the analyte concentrations of the calibrators with the analytes' peak area ratios. Fig. 2 displays the calibration curve of phenylalanine, while ESI 01† showcases the others.

#### 3.2.3 Accuracy

**Fig. 3:**
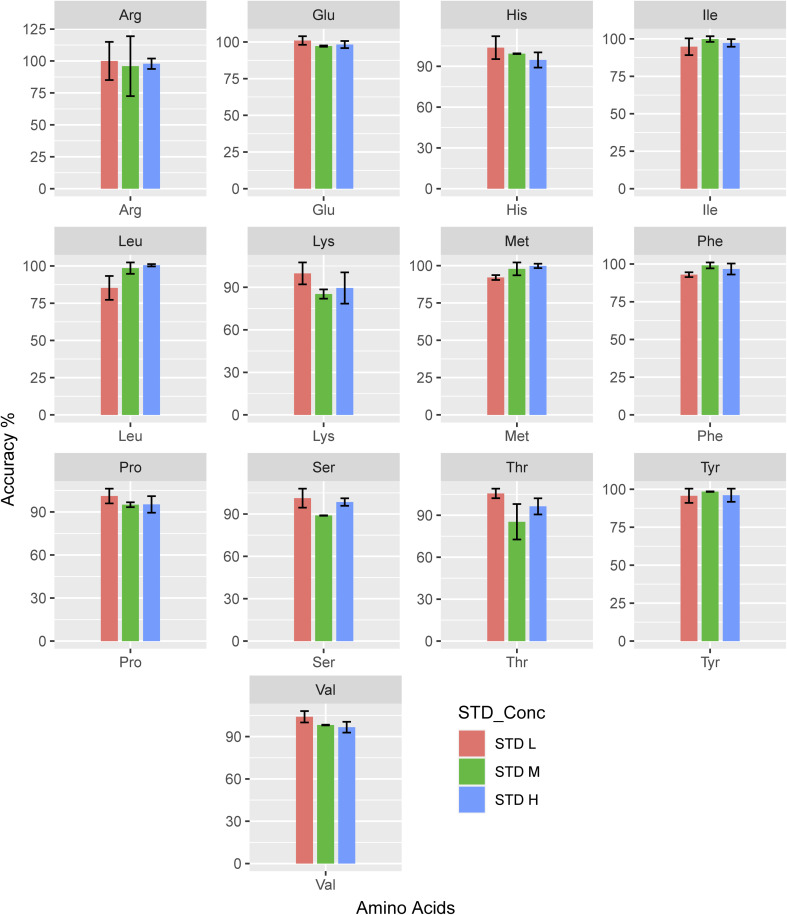
Accuracy of each amino acid's analysis (*n* = 3).

With six repetitions, the accuracy of every AA in concentrations of low (5 μM), medium (20 μM), and high (50 μM) ranged from 98.67% to 104.46% (Fig. 3). In contrast, Qu *et al.* reported their accuracy as 80.33–121.31%.^58^ Our accuracy demonstrates the method's acceptable reliability. The highest mean accuracy value is 105.63 for STD-L of threonine, whereas the lowest mean accuracy value is 92.07 for STD-L of methionine. On average, low concentration achieved a higher accuracy (98.25) than medium and high concentrations.

#### 3.2.4 Recovery

**Fig. 4:**
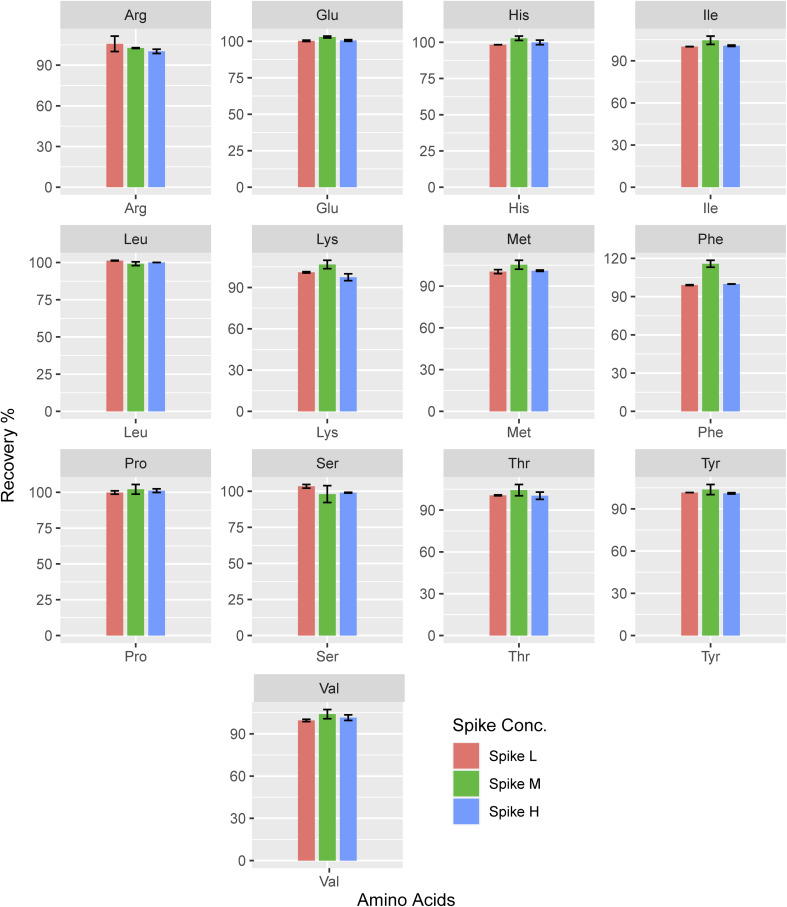
Recovery for the analysis of each amino acid (*n* = 3).

The overall average recovery of every AA at concentrations of low (5 μM), medium (20 μM), and high (50 μM) ranging from 85.039% to 109.078% (Fig. 4) showed an acceptable degree of trueness with the approach. Furthermore, all analytes showed adequate linearity in recoveries concerning concentration. The overall recoveries noted for the amino acids under investigation fell within the 85–105% range, following the AOAC Guideline for Standard Method Performance Requirements and the FDA Guidelines^48^ for validating liquid chromatographic chemical procedures.

Whereas Cheng *et al.* reported, 95.4–106.2% ,^27^ and Qu *et al.* reported 80–120% recoveries,^58^ which were in the acceptable range. However, the Trp recovery noted in the same study falls short of the acceptable threshold shown in Fig. 4. The existing data on LCMS/MS recovery tests conducted on the saliva matrix is highly sparse. For Spike-M of lysine, the highest mean accuracy value is 106.71, whereas for Spike-H of lysine, the lowest is 97.46. Compared to high and low concentrations, medium concentrations produced an accuracy that was, on average, higher (104.01). Here lysine showed higher recovery than the other amino acids and its recovery (%) is almost similar to the three spike concentrations.

#### 3.2.5 Precision

**Fig. 5:**
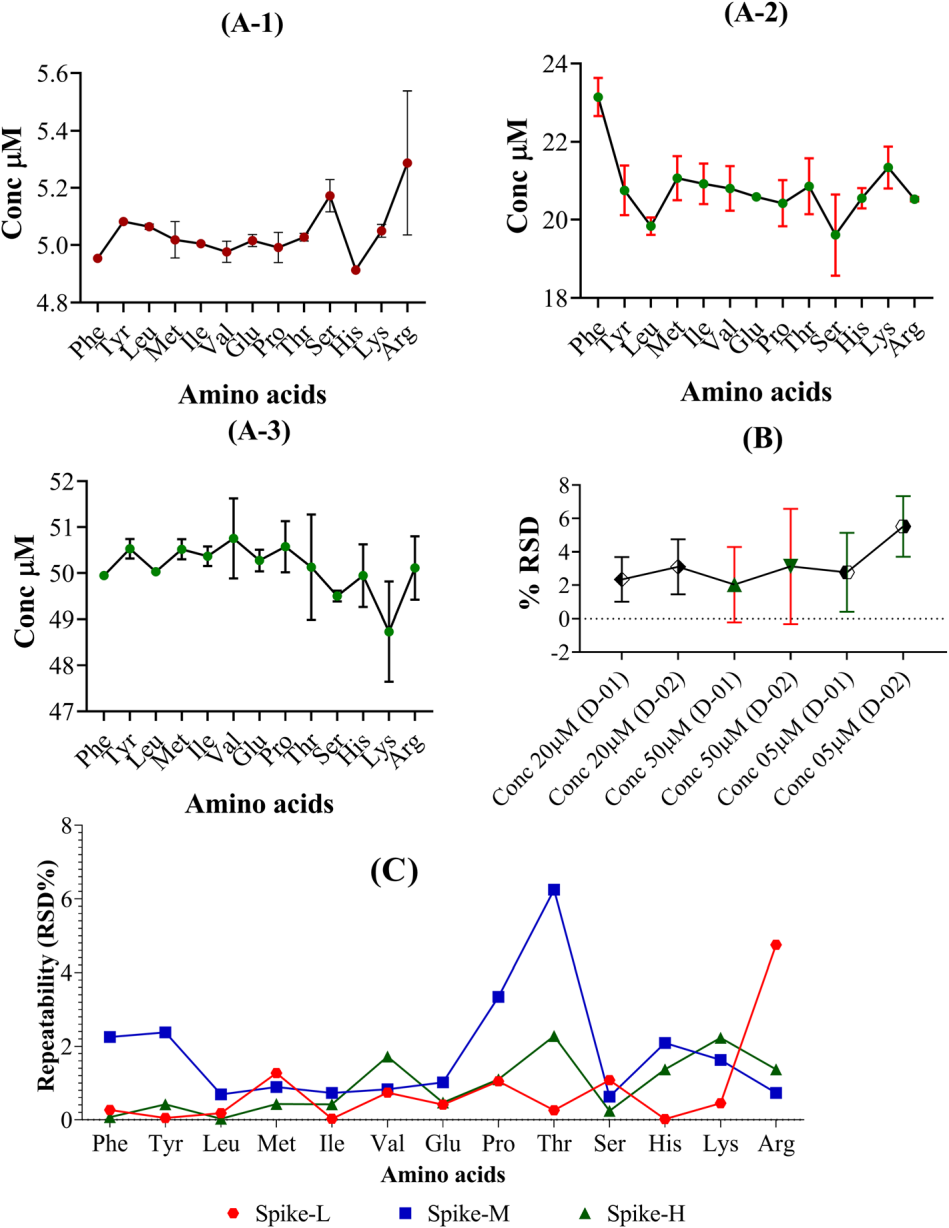
(A) Intraday (A-1-Spike-L, A-2-Spike-M, and A-3-Spike-H) and (B) interday (intermediate) precision to examine all amino acids individually. (C) Repeatabilities of amino acids at low, medium, and high concentrations of spike samples.

The percentage comparative standard deviation (% RSD) of concentrations beneath 10% for each amino acid examined indicates the method's precision, as illustrated in Fig. 5.

**Fig. 6:**
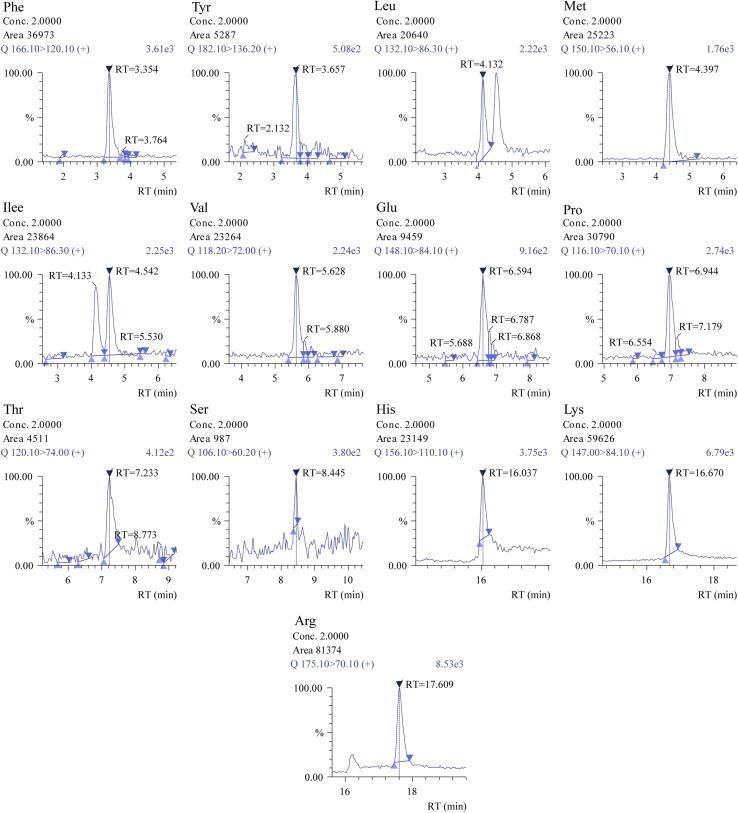
Chromatograms of compounds of interest obtained using the Shimadzu insight software. Analysis was performed using 2 μM concentration (other amino acids in ESI 02†) for the comparative study of targeted amino acids.

The % RSD values for the specific analyte concentration are 11% for repeatability and 16% for reproducibility. Fig. 5 shows the performance results for this method in terms of intra- and inter-day accuracies and precisions for simultaneous analysis of 20 AAs at four distinct concentration levels. All analytical AAs had precision values between −7.6 and 9.4% and accuracy values between −10.1% for intra- and inter-day assessments. Cheng *et al.* reported their intra-day were all <5% and the inter-day were all <7% (ref. 27) and Qu *et al.* reported that their intra-day precision ranged from 0.32–14.05%, whereas the inter-day precision ranged from 1.03–14.81%,^58^ whereas our precision is in the acceptable range (Fig. 6).

The technique exhibited acceptable reproducibility, as evidenced by the repeatability of RSD for the STD-L, STD-M, and STD-H (*n* = 6), which ranged from 0.02% to 6.25%. In contrast, Qu *et al.* demonstrated that their repeatability ranged from 0.23–9.91%,^58^ indicating acceptable repeatability for the method (Fig. 5). These findings suggest that the target AAs may be measured with accuracy, dependability, and reproducibility using the LC-MS/MS approach.

#### 3.2.6 Specificity and selectivity

A method achieves selectivity when its result differs from all other responses. It implies that the analytical technique must be able to distinguish between the molecules that are naturally present in the matrix and other sample constituents that are the analytes of interest. MRM for this method is precise for this amino acid in the experiments. The method employed methanol instead of acetonitrile, demonstrating more excellent selectivity for acetonitrile over methanol.

### 3.3 Amino acid profiling in different matrices

LC-MS/MS was utilized to analyze human saliva, fertilizer, trizepatide (pharmaceutical product), plant growth hormone (PGH), mud crab, animal feed, and fish feed samples using the established, validated technique for measuring amino acids randomly, and the recovery (%) ranged from 82% to 107%, as presented in Table 3. We found that the recoveries in human saliva were 88% to 103% (*n* = 4). On the other matrix, such as fertilizer, recoveries were 88% to 110% (*n* = 6), whereas trizepatide, a pharmaceutical product, showed 77% to 117% recoveries. We applied our validated method to animal feed (*n* = 5) and fish feed (*n* = 3), and recoveries of 75% to 93% and 82% to 107%, respectively, were obtained. As a matrix, mud crab (*n* = 3) showed recoveries of 79% to 101%. In plant growth, hormone recoveries are low, at 74% to 89%.

**Table 3:** Amino acid concentrations (%) in different matrices with their recoveries (%)

Matrix	Amino acid amount (%)													Recovery (%)
	Phe	Tyr	Leu	Met	Ile	Val	Glu	Pro	Thr	Ser	His	Lys	Arg	
Human saliva	6.38	8.70	3.88	0.80	0.80	1.56	4.90	8.25	4.10	6.14	5.38	3.60	5.71	90–94
	5.00	6.53	3.58	0.81	0.05	1.11	3.20	10.19	4.20	8.17	4.24	6.81	8.99	88–91
	3.88	3.18	3.44	0.81	0.04	1.09	2.20	3.43	4.90	9.19	3.47	3.70	3.29	93–96
	5.10	5.75	3.81	0.82	0.27	2.33	1.75	4.17	3.89	5.79	3.28	4.37	7.57	95–103
Fertilizer	0.10	0.04	0.10	0.02	0.13	0.08	0.06	0.09	0.014	0.01	0.004	0.02	0.02	92–97
	0.71	0.04	0.45	0.08	0.31	0.38	0.11	2.15	0.0003	0.01	0.02	0.13	0.13	88–95
	4.01	8.25	5.68	0.00	5.34	0.49	8.05	6.93	1.31	9.38	5.87	6.34	1.77	89–92
	5.47	6.64	4.42	0.23	7.00	1.89	0.34	7.77	0.18	5.13	6.54	8.63	7.30	95–106
	5.57	6.46	8.01	0.005	4.00	1.95	10.88	8.17	4.51	10.11	0.004	5.76	0.12	95–110
	7.38	4.15	2.63	0.00	4.57	5.11	16.99	0.04	5.60	6.81	2.07	3.62	7.48	95–109
Trizepatide (pharmaceutical product)	0.12	0.12	0.12	0.65	0.12	0.006	0.33	0.09	0.21	0.16	0.08	0.23	0.08	78–95
	0.47	0.21	0.35	0.15	0.56	0.47	1.70	0.37	0.42	0.49	0.15	0.68	0.89	89–117
	0.02	0.01	0.02	0.005	0.03	0.02	1.60	0.02	0.009	0.02	0.00	0.01	0.00	77–95
Plant GH	0.003	0.001	0.01	0.0003	0.01	0.005	0.003	0.009	0.001	0.001	0.0004	0.002	0.01	74–89
Mud crab	1.24	0.14	3.26	0.13	1.47	0.51	0.61	1.81	0.21	0.08	0.0002	0.12	0.07	96–99
	0.18	0.14	0.44	0.01	0.25	0.14	0.25	0.30	0.06	0.04	0.0006	0.04	0.09	95–101
	0.27	0.22	0.62	0.03	0.30	0.30	0.68	0.73	0.06	0.04	0.001	0.04	0.06	79–95
Animal feed	0.01	0.002	0.03	0.001	0.01	0.02	0.03	0.05	0.004	0.00	0.11	0.18	0.06	86–95
	0.01	0.00	0.01	0.001	0.01	0.01	0.04	0.08	0.10	0.00	0.07	0.51	0.08	87–93
	0.36	0.17	0.74	0.19	0.50	0.43	0.78	0.26	0.19	0.63	0.36	0.89	0.91	94–101
	0.19	0.08	0.34	0.10	0.23	0.19	0.45	0.00	0.10	0.00	0.44	0.77	0.69	91–99
	0.32	0.15	0.59	0.22	0.43	0.39	0.64	0.17	0.18	0.62	0.39	0.70	0.74	95–103
Fish feed	0.33	0.10	0.50	0.93	0.42	0.35	0.76	0.69	0.23	1.83	0.12	0.38	0.39	82–89
	0.09	0.04	0.21	0.04	0.13	0.10	0.36	0.09	0.05	0.00	0.39	0.72	0.69	75–82
	0.23	0.28	0.15	0.19	0.15	0.12	0.18	0.11	0.12	0.09	0.15	0.18	0.25	92–93

## 4. Conclusions

We have developed a simple, fast, reproducible, and non-derivatization bioanalytical approach based on LC-MS/MS to quantify 13 essential amino acids with satisfactory separation in human saliva. This method has comparatively higher specificity and sensitivity. The majority of AAs had relatively high saliva levels, according to reports.^59–64^ The LOQs of the suggested approach could fulfill most of the quantitative sensitivities. Our successful development and validation of the technology have enabled us to estimate salivary amino acids. The primary benefit of this method is its capacity to screen large batches of samples with high throughput. Our chromatographic approach made it possible to separate isomeric amino acids such as leucine and isoleucine. Compared to routine solution analysis, the surrogate matrix closely parallels human saliva. The procedure complied with the standards outlined in the regulatory guidelines of the FDA^48^ and EMA.^47^ Its simplicity, speed, and ease of use make it an excellent fit for clinical translational applications.

Additionally, it might offer an alternate technique for analyzing AAs in various complicated biological substances, including fertilizer, trizepatide (pharmaceutical product), plant growth hormone (PGH), mud crab, animal feed, and fish feed. The main focus of this work is to develop and validate a method for SFAA using the LC-MS/MS technique. We validated this method using saliva from healthy individuals rather than the case samples. It would be more fruitful if future studies engaged samples from both cases and controls to establish potential metabolic biomarkers in different disease conditions.

## Ethical clearance

Ethical clearance was approved by the Institutional Review Board of BSMMU, with reference number BSMMU/2021/7214, for the analysis of human saliva samples by LCMS/MS.

## Author contribution

Md. Mehedi Hasan: methodology, validation, formal analysis, data curation, writing – original draft Mamudul Hasan Razu: conceptualization, validation, formal analysis, investigation, project administration, writing – review and editing Sonia Akter: validation, formal analysis, data curation Salma Akter Mou: validation, formal analysis, data curation Minhazul Islam: formal analysis, visualization, writing – review and editing Mala Khan: supervision, methodology, writing – review and editing, investigation, resources, funding acquisition.

## Conflicts of interest

The authors are all employed by BRiCM. The authors do not have any further financial relationships or affiliations with organizations or entities with financial stakes or conflicts of interest with the topics or materials covered in the article except those already indicated.

## Supplementary Material

RA-014-D4RA01130A-s001
