# Supplementary material for: Development and validation of a non-invasive method for quantifying amino acids in human saliva
Source: RSC Adv. 2024 Jul 15;14(31):22292–303. doi: 10.1039/d4ra01130a (PMC11247435; doi:10.1039/d4ra01130a)
Supplement: RA-014-D4RA01130A-s001 [file RA-014-D4RA01130A-s001.pdf]

Electronic Supplementary Material (ESI) for RSC Advances.
This journal is © The Royal Society of Chemistry 2024

MS Summary(Compound)
Amino Acid_2uM_02.lcd

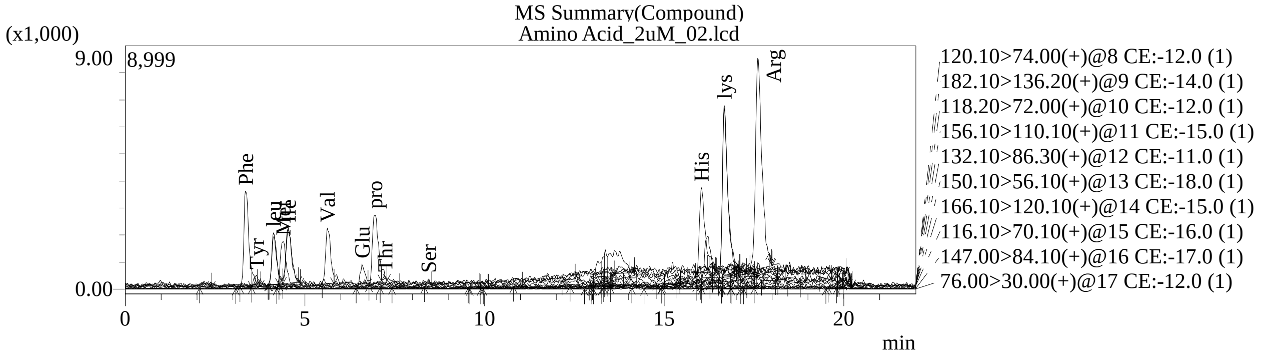

ID#1 Compound Name: Phe m/z: 166.10>120.10

Title	Ret. Time	Area	Height	Conc.
Amino Acid_2uM_02.lcd	3.354	34579	3484	1.870
Average	3.354	34579	3484	1.870
%RSD	--	--	--	--
Maximum	3.354	34579	3484	1.870
Minimum	3.354	34579	3484	1.870
Standard Deviation	--	--	--	--

ID#2 Compound Name: Tyr m/z: 182.10>136.20

Title	Ret. Time	Area	Height	Conc.
Amino Acid_2uM_02.lcd	3.657	4631	468	1.752
Average	3.657	4631	468	1.752
%RSD	--	--	--	--
Maximum	3.657	4631	468	1.752
Minimum	3.657	4631	468	1.752
Standard Deviation	--	--	--	--

ID#3 Compound Name: leu m/z: 132.10>86.30

Title	Ret. Time	Area	Height	Conc.
Amino Acid_2uM_02.lcd	4.132	16533	1874	1.602
Average	4.132	16533	1874	1.602
%RSD	--	--	--	--
Maximum	4.132	16533	1874	1.602
Minimum	4.132	16533	1874	1.602
Standard Deviation	--	--	--	--

ID#4 Compound Name: Met m/z: 150.10>56.10

Title	Ret. Time	Area	Height	Conc.
Amino Acid_2uM_02.lcd	4.397	20424	1717	1.619
Average	4.397	20424	1717	1.619
%RSD	--	--	--	--
Maximum	4.397	20424	1717	1.619
Minimum	4.397	20424	1717	1.619
Standard Deviation	--	--	--	--

ID#5 Compound Name: Ile m/z: 132.10>86.30

Title	Ret. Time	Area	Height	Conc.
Amino Acid_2uM_02.lcd	4.542	21966	1984	1.841
Average	4.542	21966	1984	1.841
%RSD	--	--	--	--
Maximum	4.542	21966	1984	1.841
Minimum	4.542	21966	1984	1.841
Standard Deviation	--	--	--	--

ID#6 Compound Name: Val m/z: 118.20>72.00

Title	Ret. Time	Area	Height	Conc.
Amino Acid_2uM_02.lcd	5.628	23813	2095	2.047
Average	5.628	23813	2095	2.047
%RSD	--	--	--	--
Maximum	5.628	23813	2095	2.047
Minimum	5.628	23813	2095	2.047
Standard Deviation	--	--	--	--

ID#7 Compound Name: Glu m/z: 148.10>84.10

Title	Ret. Time	Area	Height	Conc.
Amino Acid_2uM_02.lcd	6.594	8260	820	1.746
Average	6.594	8260	820	1.746
%RSD	--	--	--	--
Maximum	6.594	8260	820	1.746
Minimum	6.594	8260	820	1.746
Standard Deviation	--	--	--	--

ID#8 Compound Name: pro m/z: 116.10>70.10

Title	Ret. Time	Area	Height	Conc.
Amino Acid_2uM_02.lcd	6.944	29836	2531	1.938
Average	6.944	29836	2531	1.938
%RSD	--	--	--	--
Maximum	6.944	29836	2531	1.938
Minimum	6.944	29836	2531	1.938

Title	Ret. Time	Area	Height	Conc.
Standard Deviation	--	--	--	--

ID#9 Compound Name: Thr m/z: 120.10>74.00

Title	Ret. Time	Area	Height	Conc.
Amino Acid_2uM_02.lcd	7.233	4509	364	1.999
Average	7.233	4509	364	1.999
%RSD	--	--	--	--
Maximum	7.233	4509	364	1.999
Minimum	7.233	4509	364	1.999
Standard Deviation	--	--	--	--

ID#10 Compound Name: Ser m/z: 106.10>60.20

Title	Ret. Time	Area	Height	Conc.
Amino Acid_2uM_02.lcd	8.445	929	228	1.882
Average	8.445	929	228	1.882
%RSD	--	--	--	--
Maximum	8.445	929	228	1.882
Minimum	8.445	929	228	1.882
Standard Deviation	--	--	--	--

ID#11 Compound Name: His m/z: 156.10>110.10

Title	Ret. Time	Area	Height	Conc.
Amino Acid_2uM_02.lcd	16.037	22984	2704	1.986
Average	16.037	22984	2704	1.986
%RSD	--	--	--	--
Maximum	16.037	22984	2704	1.986
Minimum	16.037	22984	2704	1.986
Standard Deviation	--	--	--	--

ID#12 Compound Name: lys m/z: 147.00>84.10

Title	Ret. Time	Area	Height	Conc.
Amino Acid_2uM_02.lcd	16.670	58385	6087	1.958
Average	16.670	58385	6087	1.958
%RSD	--	--	--	--
Maximum	16.670	58385	6087	1.958
Minimum	16.670	58385	6087	1.958
Standard Deviation	--	--	--	--

ID#13 Compound Name: Arg m/z: 175.10>70.10

Title	Ret. Time	Area	Height	Conc.
Amino Acid_2uM_02.lcd	17.609	89840	7586	2.208
Average	17.609	89840	7586	2.208
%RSD	--	--	--	--
Maximum	17.609	89840	7586	2.208
Minimum	17.609	89840	7586	2.208
